# Intralipid as a matrix additive for evaluating hyperlipidemic postmortem blood

**DOI:** 10.1093/jat/bkad025

**Published:** 2023-05-02

**Authors:** Emily Elenstål, Henrik Green, Robert Kronstrand, Albert Elmsjö

**Affiliations:** Department of Forensic Genetics and Forensic Toxicology, National Board of Forensic Medicine, Artillerigatan 12, Linköping SE-58758, Sweden; Department of Forensic Genetics and Forensic Toxicology, National Board of Forensic Medicine, Artillerigatan 12, Linköping SE-58758, Sweden; Division of Clinical Chemistry and Pharmacology, Department of Biomedical and Clinical Sciences, Faculty of Medicine and Health Sciences, Linköping University, Linköping SE-581 83, Sweden; Department of Forensic Genetics and Forensic Toxicology, National Board of Forensic Medicine, Artillerigatan 12, Linköping SE-58758, Sweden; Division of Clinical Chemistry and Pharmacology, Department of Biomedical and Clinical Sciences, Faculty of Medicine and Health Sciences, Linköping University, Linköping SE-581 83, Sweden; Department of Forensic Genetics and Forensic Toxicology, National Board of Forensic Medicine, Artillerigatan 12, Linköping SE-58758, Sweden

## Abstract

Postmortem whole blood samples can differ greatly in quality where hyperlipemia is a frequent variable that can influence the results of analytical methods. The aim of this study was to investigate the influence of lipemia on postmortem analysis as well as demonstrate the usage of Intralipid in comparison to pooled postmortem lipids as matrix additives for meaningful evaluation and validation of hyperlipidemic postmortem samples. Hyperlipidemic blood samples were simulated by adding different concentrations of Intralipid or pooled authentic postmortem lipids to bovine whole blood. The hyperlipidemic blood samples were spiked with 14 benzodiazepines and five sedative and antianxiety drugs (alprazolam, clonazepam, 7-aminoclonazepam, diazepam, flunitrazepam, 7-aminoflunitrazepam, hydroxyzine, lorazepam, midazolam, nitrazepam, 7-aminonitrazepam, nordazepam, oxazepam, propiomazine, dihydropropiomazine, temazepam, triazolam, zolpidem and zopiclone). Samples were prepared with liquid-liquid extraction followed by ultra-high performance liquid chromatography–mass spectrometry. The effects of lipemia on the recovery of analytes and internal standards (ISs) were evaluated to determine the effect of, and any differences between, the two additives. Lipemia was found to cause major interference when quantifying the analytes. For most analytes, the ISs could compensate for analyte losses. However, the most hydrophilic analytes (7-amino metabolites), together with the most lipophilic analytes (propiomazine and dihydropropiomazine), were greatly affected by lipemia (<50% recovery), and the IS could not compensate for analyte losses. In general, lower analyte recoveries were observed for samples with Intralipid as a lipemic additive in comparison to those containing pooled postmortem lipids. Both Intralipid and pooled postmortem lipids showed marked effects on the analytical results. Intralipid gave a good indication of the effects of lipemia and could be a useful tool for making a meaningful evaluation of hyperlipidemic postmortem samples during the method development and validation.

## Introduction

Hyperlipemia, which could be defined as sample turbidity due to the accumulation of lipoprotein particles, is a relatively frequently reported feature of blood samples collected for analysis in the living (1). Lipemia can be caused by several factors; however, it is usually observed in a too short time elapsed between the last meal consumed and blood sampling (2). In general, a sample is considered lipemic when the triglyceride concentration is ∼3–5 mg/mL, at which concentration the plasma sample may appear turbid. However, sample turbidity and lipemia are more difficult to detect visually in whole blood samples compared to plasma (1). In liquid chromatography–mass spectrometry (LC–MS), the state of the samples can have a vast impact on the quantitative performance, and hyperlipemia has been reported to cause matrix effects through ion suppression and extraction efficiencies (3–5). According to the European Medical Agency (EMA) and the Food and Drug Administration (FDA) guidelines for bioanalytical method validation, if lipemic matrices are expected, it is recommended to investigate their effects on ion suppression, ion enhancement or extraction efficiency when using LC–MS methods (6, 7). In postmortem toxicological cases, high variability of matrices is expected and has to be taken into consideration when developing analytical methods, including those for evaluating hyperlipidemic samples. To our knowledge, there is no clear definition of a hyperlipidemic sample in postmortem toxicology, nor are there any specific recommendations on how such samples should be handled for toxicological analyses or how to validate lipemic matrices for an analytical method. The AAFS Standards Board (ASB) standard 036 describing the validation of forensic toxicological methods gives little advice but states that additional matrix samples may be needed, given the variety of sample conditions (8). Intralipid, which is a lipid emulsion used for parenteral nutrition, has been suggested as a tool for mimicking real-life lipemic samples (5, 9). The objective of this study was to investigate if Intralipid could mimic hyperlipidemic postmortem samples when evaluating the extraction recovery of commonly encountered benzodiazepines and sedative drugs in postmortem blood samples.

## Materials and Methods

### Chemicals and reference materials

Methanol (gradient grade), acetonitrile (gradient grade), 2-propanol (pro-analysis grade), ethyl acetate (reagent grade) and hydrochloric acid (pro-analysis grade) were purchased from Merck (Darmstadt, Germany); formic acid (LC–MS grade) was purchased from Fisher Scientific (Waltham, MA, USA); ammonium formate (>99%) was purchased from Sigma-Aldrich (Saint Louis, MO, USA); potassium tetraborate tetrahydrate (>99.5%) was purchased from Aldrich Chemistry (Milwaukee, WI, USA) and trichloroacetic acid (>99%) was purchased from VWR Chemicals BDH (Radnor, PA, USA). Intralipid 20% lipid emulsion (200 mg/mL) was purchased from Sigma-Aldrich.

Reference materials and internal standards (ISs) were purchased from Cerilliant (Round Rock, TX, USA; alprazolam, alprazolam-d_5_, clonazepam, clonazepam-d_4_, 7-aminoclonazepam, 7-aminoclonazepam-d_4_, diazepam, diazepam-d_5_, flunitrazepam, flunitrazepam-d_7_, 7-aminoflunitrazepam, 7-aminoflunitrazepam-d_7_, hydroxyzine, lorazepam, midazolam, nitrazepam, 7-aminonitrazepam, nordazepam, nordazepam-d_5_, oxazepam, oxazepam-d_5_, temazepam, temazepam-d_5_, triazolam, zolpidem and zopiclone), Chiron (Trondheim, Norway; propiomazine, dihydropropiomazine, zolpidem-d_6_, nitrazepam-d_5_ and 7-aminonitrazepam-d_5_) and Lipomed (Arlesheim, Switzerland; midazolam-d_4_).

### Hyperlipidemic samples

Upon arrival at the Department of Forensic Genetics and Forensic Toxicology, all samples go through a visual examination. When lipemic content causes obvious layering, turbidity or a milky appearance (Figure S1), the sample is annotated as hyperlipidemic. The degree of putrefaction is assessed individually by the forensic pathologist during autopsy. To investigate if there was a relation between putrefaction and hyperlipemia, the prevalence of different degrees of putrefaction was compared between cases annotated as hyperlipidemic (*n* ∼ 1,000) versus cases not annotated as hyperlipidemic (*n* ∼ 28,000).

In order to estimate the amount of lipids in the samples annotated as hyperlipidemic, all samples classified as hyperlipidemic during 1 month were visually inspected a second time. The proportion of the lipid layer in the refrigerated sample was compared with the water layer using a metric ruler, and the lipemic concentration was estimated from that proportion.

### Preparation of lipemic samples

To bovine blood (containing trisodium citrate dihydrate), a standard solution with 19 analytes was added, resulting in analyte concentrations of 35, 84 or 140 ng/g depending on their calibration range. To investigate if signal intensity was affected by the amount of lipids, the blood was also spiked with Intralipid 20% lipid emulsion (200 mg/mL) in duplicates at six different concentrations (0.0, 2.4, 5.6, 12, 24 and 64 mg/g; Table S1). To further evaluate Intralipid’s possibility to simulate hyperlipidemic postmortem samples, a postmortem lipid mixture (PM-lipids) was created for reference. The PM-lipids were made by collecting the upper lipid layers from seven unique cases with a negative toxicological screening analysis (lower limit of quantification were 2–25 ng/g for the investigated analytes). The PM-lipids were added in duplicates at three concentrations at 0, 8 and 52 mg/g (milligram pooled lipids per gram whole blood; Table S2). All samples were equilibrated for 5–10 min before storage (8℃).

### Analytical procedure

Blood samples were stored at 8°C until analysis (at least 24 hr). Each sample was brought to room temperature before homogenization by vortexing, and 0.5 g was moved to a new test tube. To each sample, ISs (25 µL) were added and vortexed, followed by adding 250 µL 0.6 M borate buffer at pH 9. Extraction was carried out for 5 min using ethyl acetate (1.5 mL). The organic phase was transferred to new tubes and evaporated. All samples were reconstituted in mobile phase A together with acetonitrile with 0.05% formic acid (50/50). The analysis was performed on a Waters ultra-high performance liquid chromatography–mass spectrometry system (Acquity UPLC with a Xevo TQD) using an reversed-phase ultra-performance liquid chromatography column (BEH-C18, 50 × 2.1 mm and 1.7 µm) and electrospray ionization in the positive mode. Mobile phase A was 10 mM ammonium formate with 0.05% formic acid and mobile phase B was methanol with 0.05% formic acid. The gradient started with 1% mobile phase B for 0.5 min, then a linear gradient between 20% and 40% mobile phase B for >3 min, then 40% mobile phase B for 0.5 min, then 60–70% mobile phase B for >0.75 min, followed by 95% mobile phase B for 1 min and ending with 1% mobile phase B. The injection volume was 2 µL, the flow rate was 700 µL/min, the column temperature was 60°C and the sample chamber temperature was 10°C. The capillary voltage was 0.7 kV, cone voltage was individual for each analyte, extractor voltage was 3 V and radio frequency lens voltage was 2.5 V. The source temperature was 150°C and the desolvation temperature was 650°C. The desolvation gas flow rate was 1,000 L/hr and the cone gas flow rate was 50 L/hr.

To investigate the contribution of ion suppression, post-column infusion experiments were carried out on alprazolam, 7-aminoclonazepam and propiomazine (0.5 µg/mL) using bovine blood with Intralipid concentrations at 0, 4.0, 10, 30 and 60 mg/g.

## Results

**Figure 1.**
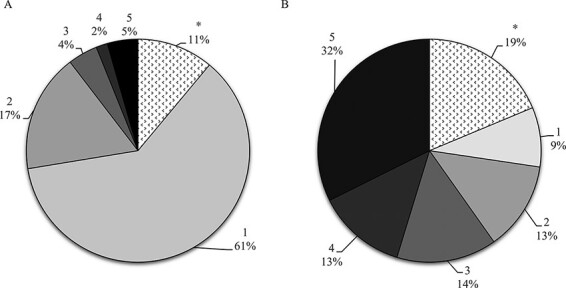
The comparison between the prevalence of different putrefaction degrees in all autopsy cases (A) and in autopsy cases with documented hyperlipemic samples (B). The degree of putrefaction is assessed on a 1–5 scale, where 1 is no putrefaction and 5 is severe putrefaction. The asterisk indicates cases with other documented observations such as skin loss, maggots and burned.

From January 2017 to December 2021, ∼28,000 autopsy cases were analyzed at the Department of Forensic Genetics and Forensic Toxicology. Out of these, 1,070 autopsy cases were classified as hyperlipidemic. The prevalence of putrefaction was vastly different in those cases with hyperlipidemic samples (Figure 1). In general, 61% of cases show no putrefaction, and only 5% present with severe putrefaction, whereas in the hyperlipidemic cases, only 9% show no putrefaction, but 32% show severe putrefaction, suggesting a correlation between putrefaction and hyperlipidemic blood samples. During the test period, the mean lipid content in whole blood was ∼5% (v/v), which roughly corresponds to 50 mg/g.

### Intralipid and postmortem lipid effect on analyte recoveries

**Figure 2.**
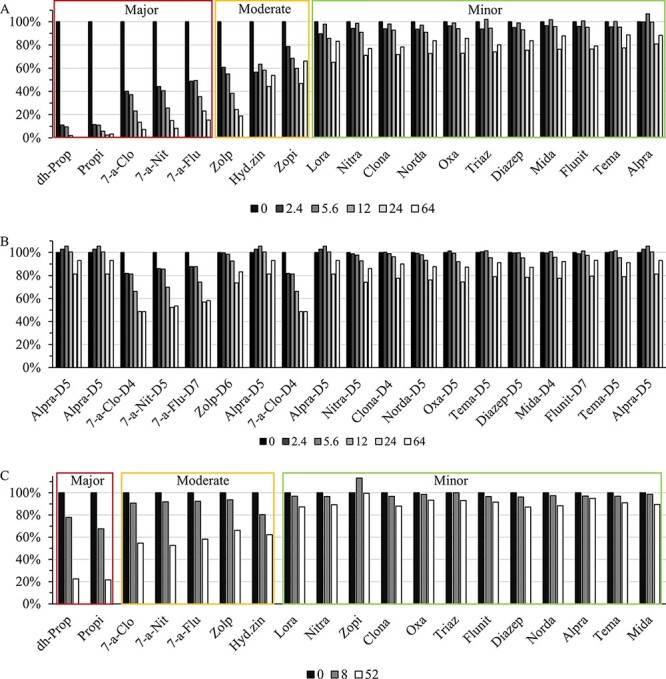
Analyte-dependent matrix effects. (A) Average analyte recoveries dependent on the amount of added Intralipid at 0, 2.4, 5.6, 12, 24 and 64 mg/g. (B) IS recoveries dependent on the amount of added Intralipid at 0, 2.4, 5.6, 12, 24 and 64 mg/g. (c) Analyte recoveries dependent on the amount of added postmortem lipids at 0, 8 and 52 mg/g. Alpra, alprazolam; Clona, clonazepam; 7-a-Clo, 7-aminoclonazepam; Diazep, diazepam; Flunit, flunitrazepam; 7-a-Flunit, 7-aminoflunitrazepam; Hyd.zin, hydroxyzine; Lora, lorazepam; Mida, midazolam; Nitra, nitrazepam; 7-a-Nit, 7-aminonitrazepam; Norda, nordazepam; Oxa, oxazepam; Propi, propiomazine; dh-Prop, dihydropropiomazine; Tema, temazepam; Triaz, triazolam; Zolp, zolpidem; Zopi, zopiclone.

Bovine whole blood was spiked with analytes together with varying amounts of lipids in order to investigate how the analytes were affected by the amount of lipids. Recoveries were calculated by dividing the absolute area from the lipemic samples with the corresponding absolute area from the reference sample without lipids added. In general, a higher lipemic content results in decreased analyte recoveries (Figure 2A).

At the hyperlipidemic concentration of 5.6 mg/g, a concentration impossible to visually determine as hyperlipidemic, the analytes could be divided into three subcategories; minorly affected analytes with recoveries >75% (alprazolam, diazepam, flunitrazepam, clonazepam, lorazepam, midazolam, nitrazepam, nordazepam, oxazepam, temazepam and triazolam), moderately affected analytes with recoveries between 75% and 50% (zopiclone, zolpidem and hydroxyzine) and majorly affected analytes with recoveries <50% (7-aminonitrazepam, 7-aminoclonazepam, 7-aminoflunitrazepam, propiomazine and dihydropropiomazine; Figure 2A). The recovery of ISs at 5.6 mg/g was between 79% and 101% (Figure 2B). The agreement between duplicates was acceptable for all analytes (0.1–11%), except for zopiclone (13–37%) and the majorly affected analytes at lipid concentrations >12 mg/g.

PM-lipids were added to samples at three concentrations at 0, 8 and 52 mg/g. Similar results as those in Intralipid investigation were observed, where increasing lipid amounts seem to decrease analyte recoveries (Figure 2C). Even though the same pattern was observed, higher analyte recoveries were generally observed with PM-lipids as a matrix additive in comparison to Intralipid. The ISs were only minorly affected, and all had an IS response >81% at the highest lipid level.

The possible contribution of negative matrix effects occurring during the ionization process was investigated with post-column infusion. Three analytes were chosen, two with major loss (propiomazine and 7-aminoclonazepam) in recovery and one with only minor loss (alprazolam). No apparent ion suppression was observed for alprazolam, 7-aminoclonazepam or propiomazine. The signal intensities from the post-column infusion experiment of propiomazine for a blank, a non-lipidemic whole blood sample and an extremely lipidemic whole blood sample are shown in Figure S2.

## Discussion

Hyperlipidemia can be defined as the turbidity of the sample caused by the accumulation of lipoprotein particles. The overall frequency of hyperlipidemic samples in clinical settings ranges from 0.5% to 2.5% (10). According to the European Bioanalysis Forum, the validation of hyperlipidemic plasma samples should be carried out at least at 3 mg triglycerides per milliliter plasma, which corresponds to where a plasma sample starts to appear turbid (9). In whole blood, hyperlipidemia is harder to detect visually, and the turbidity is usually not noticeable in concentrations <10 mg/mL. For postmortem samples, hyperlipidemia is probably even harder to detect due to the biochemical changes the postmortem interval creates. According to Ondruschka et al., the grade of lipemia increases with postmortem interval (11), and according to our results, the degree of putrefaction also contributes to the prevalence of hyperlipidemia, further demonstrating the importance of investigating the effect of hyperlipidemic samples in postmortem toxicology. In contrast to the FDA and EMA guidelines for bioanalytical validation, the ASB standard 036 does not mention hyperlipidemic samples as a specific condition but instead states that additional matrix samples may be needed to give a variety of sample conditions (8). Tools to investigate and validate the effects of hyperlipidemic samples in postmortem toxicology are therefore needed.

### Hyperlipemia and matrix effects

High lipid levels are often associated with an increased risk of matrix effects such as ion suppression or affected extraction recoveries (12). When working with isotopic labeled IS, matrix effects are often disregarded as it is believed that the ISs will fully compensate for potential analyte losses due to ion suppression or extraction recoveries. This study clearly demonstrates that hyperlipidemia can cause matrix effects, in turn affecting the quantification of the investigated analytes in postmortem samples. For the analytes that were moderately and majorly affected, four out of seven have their own stable isotopic labeled IS. The results demonstrate that the isotopic labeled ISs for 7-amino metabolites and zolpidem cannot compensate for analyte losses (Figure 2). Surprisingly, both post-column infusion experiments (Figure S2) and the recovery of ISs during these experiments showed no drastic loss in recoveries compared to the analytes. These results demonstrate that isotopic labeled ISs are not always able to compensate for analyte losses. These results imply that the matrix effects occur before the ionization process and might respond to a time-dependent “loss” in analytical recovery from the sample matrix. Interestingly, the most lipophilic molecule, propiomazine, with a Log *P* value of 4.8 (PubChem, XLogP3 3.0) showed the most extensive analyte losses. This indicates that lipophilic analytes partition the lipid particles to a higher extent during the pre-analytical steps, which causes the observed matrix effects and loss in recoveries (1). One reason why the ISs were unable to compensate for analyte losses could be that the high lipemic content causes difficulties with homogenization and that the time for equilibrating the samples with the ISs should be increased.

The instability of analytes could also explain the results. However, by comparing the samples to a concurrent reference sample that did not have lipids added, any potential stability bias was accounted for and normalized. In addition, there are several publications about these substances’ stability in postmortem blood, and all substances under investigation except for zopiclone seem to be stable for 2 weeks if refrigerated (13). Zopiclone’s instability may also explain the inconsistency between duplicate analyses (14).

### Intralipid and pooled postmortem lipids as matrix additives

Both Intralipid and the PM-lipids demonstrate decreased analyte recoveries with an increasing amount of lipids, and both additives identify which analytes are affected the most by hyperlipemia. Surprisingly, there was a large difference in the actual decrease in recoveries between the two additives. For example, propiomazine had an analyte recovery of ∼2% in samples spiked with Intralipid, while the recovery was ∼20% in samples spiked with PM-lipids. Intralipid’s ability to mimic authentic samples has been questioned, as Intralipid does not cover as large part of the lipid variation as authentic lipids (9, 15). Instead, hyperlipidemic samples from high triglyceride donors have been suggested. Spiking with PM-lipids represents an attempt to create “authentic samples” and was primarily used to investigate the potential issue with lipemic samples. As this procedure might be difficult to standardize due to variations that may exist between cases and samples, a more quantitative and controlled approach such as using Intralipid may be preferred. In addition, Intralipid has the benefit that it is guaranteed drug-free, minimizing the risk of contamination from the authentic samples used and enabling the addition of a specific amount of lipids in the hyperlipidemic sample. The last parameter is of high importance when mimicking postmortem lipemia as the lipid concentration might be biologically unfeasible to reach with triglyceride donors. Furthermore, the lower recovery observed for samples spiked with Intralipid in comparison to PM-lipids could be beneficial from a validation perspective as it may indicate that if the extraction procedure can recover the compounds from Intralipid, it is likely to do so in lipemic postmortem samples as well.

## Conclusion

This study shows that the matrix effects caused by hyperlipemia can vary even between analytes that have similar chemical structures and functional groups. In general, increased lipid content led to decreased analyte responses, for which the isotopic labeled ISs were not able to compensate. These matrix effects seemed to relate primarily to poor extraction recovery and were not an effect of ion suppression. Differences were observed between Intralipid and pooled postmortem lipids as matrix additives; however, both additives indicate which analytes seemed to be most affected by hyperlipemia.

Hyperlipidemia, although not under the spotlight within postmortem toxicology, can be a significant source of laboratory errors, and each forensic toxicology lab should be aware of the problem it can cause. To our knowledge, this is the first study evaluating matrix additives for creating hyperlipidemic postmortem samples. We suggest that each lab should have written procedures for evaluating and validating hyperlipidemia, where Intralipid could be used as a simple matrix additive for evaluating hyperlipidemic samples.

## Supplementary Material

bkad025_Supp

## Data Availability

The data underlying this article will be shared on reasonable request to the corresponding author.
