# Supplementary material for: Intralipid as a matrix additive for evaluating hyperlipidemic postmortem blood
Source: J Anal Toxicol. 2023 May 2;47(6):529–34. doi: 10.1093/jat/bkad025 (PMC10362951; doi:10.1093/jat/bkad025)
Supplement: bkad025_Supp [file bkad025_supp.zip › jat-22-3892-File002.docx]

Supplemental


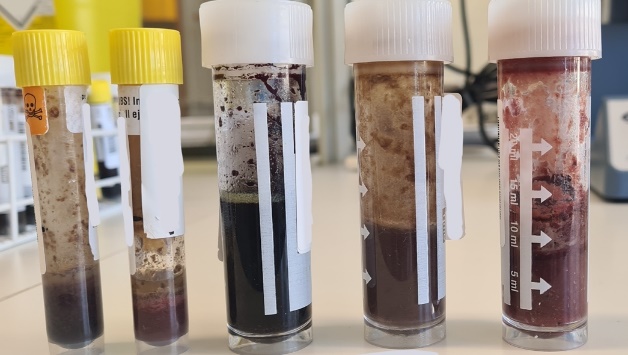


Figure S1. Authentic hyperlipemic whole blood samples.


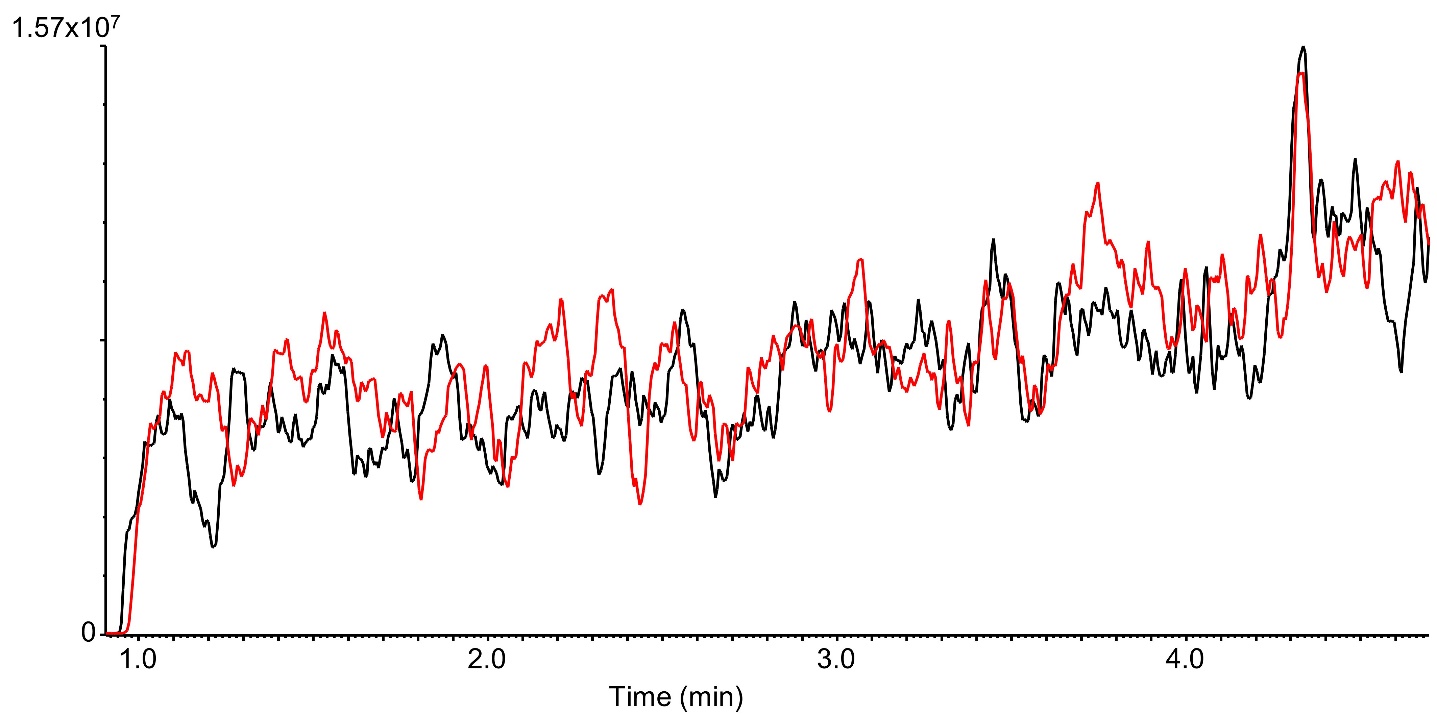
Figure S2. Chromatogram from a post-column infusion experiment of propiomazine when injecting a blank (red) and a hyperlipemic sample at 60 mg/g (black).

Table S1. Sample spiking scheme for lipemic concentration dependence experiment.

| Lipemic concentration | Volume IS solution | Volume Intralipid  (200 mg/ml) | Volume blank blood |
| --- | --- | --- | --- |
| Reference – 0.0 mg/g | 25 µL | - | 2.50 g |
| Low ­– 2.4 mg/g | 25 µL | 30 µL | 2.47 g |
| Intermediate – 5.6 mg/g | 25 µL | 70 µL | 2.43 g |
| High – 12 mg/g | 25 µL | 150 µL | 2.35 g |
| Very High – 24 mg/g | 25 µL | 300 µL | 2.20 g |
| Extreme – 64 mg/g | 25 µl | 800 µL | 1.70 g |

Table S2. Sample spiking scheme for postmortem lipids.

| Lipemic concentration | Volume IS solution | Postmortem lipids | Volume blank blood |
| --- | --- | --- | --- |
| Reference – 0.0 mg/g | 25 µL | - | 2.50 g |
| Low – 8 mg/g | 25 µL | 20 mg | 2.48 g |
| High – 52 mg/g | 25 µL | 130 mg | 2.37g |

| Table S3. Analyte and internal standard recoveries after spiking with Intralipid | | | | | | | |
| --- | --- | --- | --- | --- | --- | --- | --- |
|  | **Analyte** | **0 mg/ml** | **2.2 mg/ml** | **5.6 mg/ml** | **12 mg/ml** | **24 mg/ml** | **64 mg/ml** |
| Major | dh-Prop | 100% | 11% | 10% | 2% | 0% | 0% |
|  | Propi | 100% | 11% | 11% | 6% | 2% | 3% |
|  | 7-a-Klo | 100% | 40% | 37% | 23% | 14% | 7% |
|  | 7-a-Nit | 100% | 44% | 41% | 26% | 15% | 8% |
|  | 7-a-Flu | 100% | 49% | 49% | 36% | 23% | 15% |
| Moderate | Zolp | 100% | 61% | 55% | 38% | 24% | 19% |
|  | Hyd.zin | 100% | 57% | 63% | 58% | 44% | 54% |
|  | Zopi | 100% | 79% | 68% | 60% | 47% | 66% |
| Minor | Lora | 100% | 90% | 98% | 86% | 65% | 83% |
|  | Nitra | 100% | 94% | 99% | 91% | 71% | 77% |
|  | Klona | 100% | 94% | 98% | 93% | 72% | 78% |
|  | Norda | 100% | 94% | 97% | 91% | 73% | 84% |
|  | Oxa | 100% | 96% | 99% | 94% | 73% | 86% |
|  | Triaz | 100% | 94% | 102% | 94% | 74% | 80% |
|  | Diazep | 100% | 95% | 99% | 93% | 75% | 84% |
|  | Mida | 100% | 97% | 102% | 96% | 76% | 88% |
|  | Flunit | 100% | 96% | 101% | 95% | 76% | 79% |
|  | Tema | 100% | 96% | 100% | 95% | 77% | 89% |
|  | Alpra | 100% | 100% | 107% | 100% | 81% | 88% |
| Internal standards | Alpra-D5 | 100% | 103% | 105% | 100% | 81% | 93% |
|  | 7-a-Clo-D4 | 100% | 82% | 81% | 66% | 49% | 49% |
|  | 7-a-Nit-D5 | 100% | 86% | 86% | 70% | 52% | 53% |
|  | 7-a-Flu-D7 | 100% | 88% | 88% | 74% | 57% | 58% |
|  | Zolp-D6 | 100% | 100% | 98% | 92% | 74% | 83% |
|  | Nitra-D5 | 100% | 99% | 98% | 93% | 74% | 86% |
|  | Klona-D4 | 100% | 100% | 99% | 96% | 78% | 90% |
|  | Norda-D5 | 100% | 99% | 98% | 93% | 76% | 88% |
|  | Oxa-D5 | 100% | 101% | 99% | 92% | 74% | 87% |
|  | Tema-D5 | 100% | 101% | 101% | 95% | 79% | 91% |
|  | Diazep-D5 | 100% | 99% | 100% | 95% | 78% | 87% |
|  | Mida-D4 | 100% | 100% | 101% | 96% | 78% | 92% |
|  | Flunit-D7 | 100% | 99% | 101% | 98% | 79% | 93% |

*Analytes are grouped by how affected they were (major, moderate and minor). Recoveries are calculated by dividing the absolute area from the lipemic samples (0, 2.2, 5.6, 12, 24, 64 mg/ml) with the corresponding absolute area from the reference sample without lipids added. The absolute areas have not been normalized with the areas of the internal standard.*


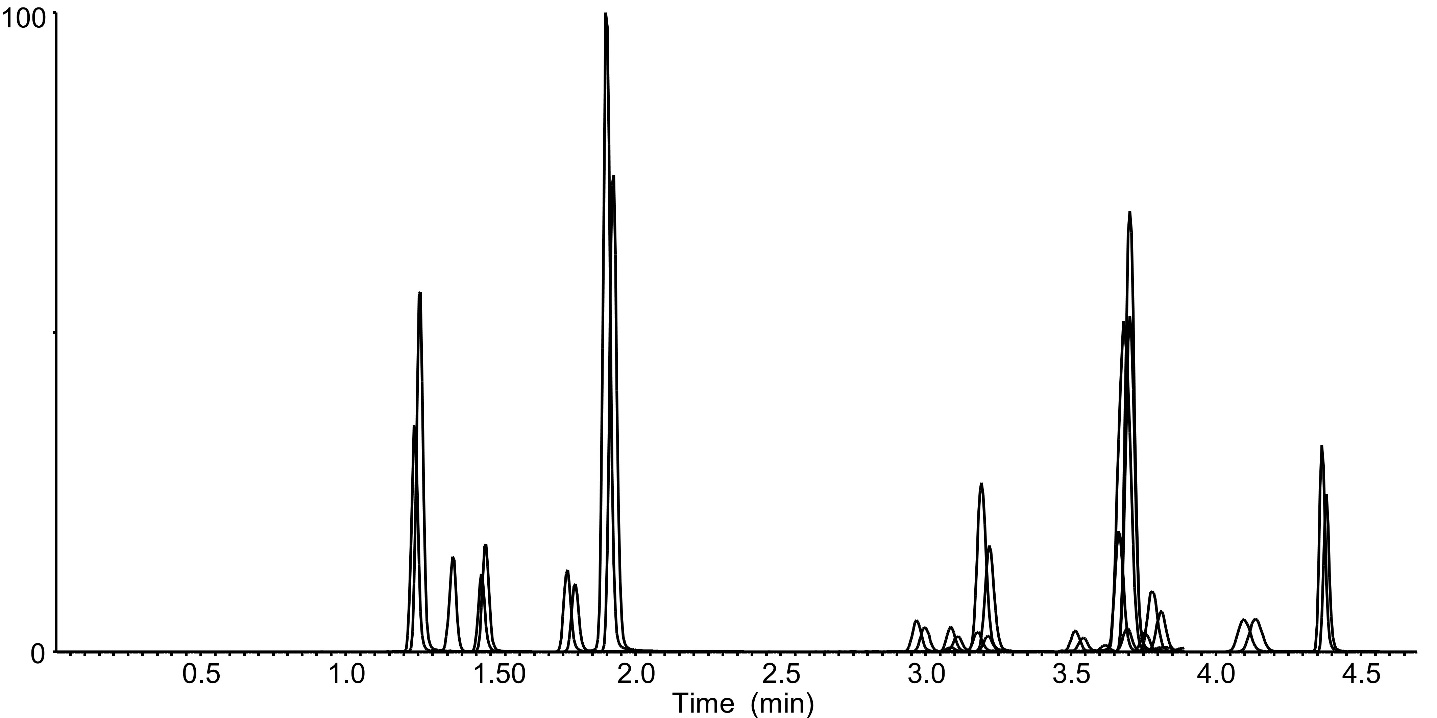
 Figure S3. Extracted ion chromatograms with one transition for each substance and internal standard. The retention order are the following: 7-aminonitrazepam 1.2, zopiclone 1.4, 7-aminoclonazepam 1.5, 7-aminoflunitrazepam 1.8, zolpidem 2.0, nitrazepam 3.0, clonazepam 3.1, flunitrazepam 3.2, midazolam 3.2, oxazepam 3.5, lorazepam 3.6, alprazolam 3.7, triazolam 3.7, dihydropropiomazine 3.7, hydroxyzine 3.8, propiomazine 3.8, temazepam 3.8, nordazepam 4.1, diazepam 4.4, 7-aminonitrazepam-D5 1.2, 77-aminoclonazepam-D4 1.4, 7-aminoflunitrazepam-D7 1.7, zolpidem-D6 1.8, nitrazepam-D5 2.9 clonazepam-D4 3.0, midazolam-D4 3.1, flunitrazepam-D7 3.2, oxazepam-D5 3.4, alprazolam-D5 3.6, temazepam-D5 3.7, nordazepam-D5 4.0 and diazepam-D5 4.3.
